# Building Enhanced Public Health Data Systems With a Situational Awareness and Learning Tool: Focus Group Study

**DOI:** 10.2196/77379

**Published:** 2026-04-29

**Authors:** Cole Brokamp, Carson S Hartlage, Tiffany Mattingly, Pierce Kuhnell, Andrew Vancil, Andrew F Beck, David Hartley

**Affiliations:** 1 Department of Pediatrics, College of Medicine, University of Cincinnati, Cincinnati, OH, United States; 2 Division of Biostatistics and Epidemiology, Department of Pediatrics, Cincinnati Children's Hospital Medical Center, 3333 Burnet Ave, Cincinnati, OH, 45229, United States, 1 513-550-0000; 3 The Health Collaborative, Cincinnati, OH, United States; 4 Division of General and Community Pediatrics, Department of Pediatrics, Cincinnati Children's Hospital Medical Center, Cincinnati, OH, United States; 5 James M Anderson Center for Health Systems Excellence, Department of Pediatrics, Cincinnati Children’s Hospital Medical Center, Cincinnati, OH, United States; 6 Division of Hospital Medicine, Department of Pediatrics, Cincinnati Children’s Hospital Medical Center, Cincinnati, OH, United States; 7 Michael A. Fisher Child Health Equity Center, Cincinnati Children’s Hospital Medical Center, Cincinnati, OH, United States; 8 Office of Population Health, Cincinnati Children’s Hospital Medical Center, Cincinnati, OH, United States

**Keywords:** data systems, public health informatics, evaluation study, public health, infectious disease

## Abstract

**Background:**

Situational awareness is the accurate and timely perception of factors in the environment, comprehension of their meanings, and projection of their future states.

**Objective:**

We aimed to develop a cloud-based Situational Awareness and Learning Tool (SALT) that generates near-real-time analytic content from multimodal health care, government, community, and environmental data, enabling public health and hospital professionals to make informed decisions during complex population health challenges.

**Methods:**

Several focus groups were conducted with representatives from local health departments, hospitals, and emergency agencies. The first round identified data needs and requirements to inform SALT’s design. SALT was developed as a secure, cloud-based platform featuring automated deployment, role-based access, and version-controlled content publishing. The second round of focus groups evaluated the SALT prototype’s utility and gathered feedback for improvements.

**Results:**

Participants highlighted the need for integrated data from multiple sources, tailored dashboards for specific audiences, and legal frameworks to guide timely data sharing. SALT met these requirements by providing interactive visuals, secure access levels, and a collaborative content management system. The second focus groups affirmed SALT’s effectiveness in enhancing decision-making and strategic planning, suggesting enhancements such as clearer data labeling, expanded data coverage, and forecasting capabilities.

**Conclusions:**

SALT addresses limitations exposed by the COVID-19 pandemic in public health data systems by offering a scalable platform for data sharing, rapid analysis, and situational awareness. It fulfills user needs for integrated, timely data, and customized analytic products. SALT represents a viable solution for enhancing public health data systems in preparation for future pandemics and other complex, multisector population health challenges.

## Introduction

Public health data systems are essential for monitoring, preventing, and responding to infectious diseases, especially those with pandemic potential. To be effective, systems should support comprehensive situational awareness: accurate and timely perception of factors affecting public health and well-being, comprehension of their meanings, and projection of key factors into the future [1]. The COVID-19 pandemic exposed the limitations and gaps in existing data systems at local, state, national, and global levels for providing adequate situational awareness. For example, in the early waves of COVID-19, many hospitals and public health agencies faced challenges in collecting, integrating, analyzing, and sharing timely and accurate data on testing, cases, hospitalizations, deaths, vaccinations, and other indicators of COVID-19 transmission and impact [2]. These challenges are broad and can be related to (1) technology and interoperability of systems, (2) access and privacy concerns, and (3) a lack of reproducible and scientific data reporting pipelines [3-5]. Such difficulties persist, placing our public health and health care systems at a significant disadvantage [6]. Moreover, many data systems lack the capacity to incorporate information on the social and environmental determinants of health. Indeed, factors such as poverty, housing, air quality, and mobility affect the vulnerability and resilience of populations to COVID-19 and other health threats [7]. As a result, public health decision-makers and partners often lack the situational awareness and learning tools needed to effectively plan, implement, and evaluate evidence-based and equity-oriented interventions to mitigate and prevent COVID-19 and future pandemics [8].

Recent work further emphasizes that these limitations extend beyond outbreak surveillance alone. Inadequate integration of social, demographic, and environmental determinants of health, including housing conditions, poverty, air quality, and mobility patterns, has constrained the ability of public health agencies to identify vulnerable populations and deploy equity-oriented interventions in real time [5,7]. National reviews of pandemic data infrastructures highlight that delays and inconsistencies in reporting materially affected operational decision-making and resource allocation, reinforcing calls for modernized, interoperable, and policy-aligned public health data ecosystems [4-6].

In response to these calls, federal agencies and public health leaders have increasingly emphasized investments in data modernization, cloud-based infrastructure, and interoperable surveillance architectures capable of supporting near-real-time analytics across clinical, community, and environmental domains [6]. Automated, reproducible platforms that harmonize multimodal data sources and disseminate tailored analytic products through role-based permissions have therefore emerged as a central component of pandemic preparedness and disaster response strategies [6,8,9].

With this backdrop, our objective was to create a situational awareness and learning tool (SALT) capable of generating near-real-time analytic content using multimodal data. We also sought to make SALT as useful as possible for frontline public health and hospital professionals, optimized in ways that allowed them to make informed decisions tuned to on-the-ground conditions.

## Methods

### Overview

SALT development drew upon our experience in the southwest Ohio COVID-19 response beginning in February 2020. During that time, we worked collaboratively with major interest holders, including hospital systems, public health, and the congregate care community, to identify data needs and then rapidly build an ad hoc situational awareness system [6-8]. When the public health emergency ended in May 2023, local interest holders expressed the need to perpetuate and expand the functionality of the legacy system beyond COVID-19.

### Design Focus Groups

A qualitative methodology [10,11] using focus groups was selected to capture nuanced, context-specific insights from stakeholders involved in the COVID-19 response. This approach facilitated interactive discussion, allowing participants to collectively reflect on data challenges and inform the user-centered design and refinement of SALT.

We conducted 2 iterative rounds of focus groups, one centered on design and the second on the evaluation of SALT, in collaboration with a nonprofit partner, The Health Collaborative. For the first round, we purposively [12] sampled leaders from various agencies and organizations involved in the pandemic response in counties within the Ohio Hospital Association’s COVID-19 Zone 6, including city and county public health departments, hospitals, and emergency management agencies (EMAs), using targeted email outreach.

Focus groups were held virtually on a video conferencing platform (Microsoft Teams) and used a semistructured interview format, with open-ended questions and prompts to guide discussion. Sessions lasted approximately 60 minutes. Each focus group was attended by at least three members of The Health Collaborative, who moderated and took field notes throughout. Sessions were audio recorded and transcribed. Following each session, the research team collaboratively reviewed notes and transcripts to discuss emergent ideas and prioritize themes. Multiple authors reviewed the outputs from the focus groups to summarize responses to specific questions and interpret similarities across different group discussions. Data were collected until no new ideas were mentioned by participants.

The first round of focus groups occurred in November 2023 and consisted of 3 sessions. The goal of these sessions was to identify data elements, sources, and information of potential benefit to ongoing COVID-19 phases and future pandemic preparedness. A standard agenda and interview guide were used for all sessions (Multimedia Appendix 1). The questions focused on organizational data access, data-informed decisions, frustrations with available data, and visions for an ideal data tool during future emergencies.

Themes from these focus groups were used to inform the initial design and functionality of SALT, with particular attention to desired data types, visualization needs, and system usability. In parallel, we compiled a list of data sources mentioned by participants and assessed each for availability, accessibility, and quality as potential inputs for the tool.

### Initial SALT Build

The first set of focus groups, and our experience during various phases of the COVID-19 pandemic [2,8,9,13-16], informed the initial SALT build. We designed the SALT architecture to automatically deploy onto a cloud infrastructure provider as a virtual private cloud (VPC). VPC offers advantages over on-premises hosting, including security, backup and failover, and extensible compute capability. To ensure high availability, we deployed SALT in 2 geographical data center regions and with fully automatic database failover in the event of a vendor hardware failure. Cloud-based computing resources are abundantly available and are dynamically allocated to the rendering or serving of content based on real-time demand. Heavily accessed items automatically receive additional resources to maintain functionality.

Access levels for SALT products include “public” (no account required), “authenticated” (account required), and “authorized” (account with product-specific permissions required). Authentication is achieved through federation with InCommon, and accounts are created by a SALT administrator after agreement to SALT policies. Content is created by SALT publishers, who create the code-based content for SALT users to consume. Authorization for specific content is managed by the content publisher and audited by SALT administrators.

To enhance the efficiency, security, and integrity of content publishing, a detailed procedure has been established for publishers. Initially, potential publishers engage with SALT administrators to evaluate the compatibility of their content for inclusion. Upon approval, publishers are required to complete a brief survey and agree to an acceptable use policy available on the SALT landing page. Following this, SALT administrators configure a user account, marking the entry into the publishing workflow.

For integration into SALT, all code used to generate content is housed within a version-controlled Git repository. Contributors without existing version control are supported in setting up a new repository within the SALT organization on GitHub. For contributors with a preexisting repository, that repository is forked into the SALT organization to ensure that any updates follow the established review and integration protocols.

Publishers initiate content deployment by logging into SALT and navigating the step-by-step deployment process. Initially, content is uploaded in a private, developmental state and subjected to a thorough review process involving automated deployment tests and code reviews by SALT administrators. This ensures that only high-quality, fully vetted content reaches production status. Following the approval, the content is made live, and the developmental version is archived, preserving the integrity and history of updates through the version-controlled repository.

Adjustments to specific content settings, such as access levels, URL customization, and scheduling of content rendering or emailing, are collaboratively managed by the publisher and SALT administrators. This meticulous process ensures that SALT continuously offers accurate, up-to-date situational awareness through a scalable and transparent system.

To deliberately uphold data privacy, SALT was designed not to store protected health information or other sensitive data within its system. Instead, it accesses necessary data sources only at the precise moment a report is generated. Processing sensitive or private data in real-time prevents the need to retain it, minimizing the risk of unauthorized access. By performing “just in time” automated data retrieval and analysis, SALT prevents human interaction with raw sensitive data. The tool exclusively shares derived analytical content, enabling informed decision-making with shared data while maintaining strict data confidentiality and compliance with privacy regulations.

### Evaluation Focus Groups

The second round of focus groups aimed to evaluate the SALT prototype and included 2 sessions held in June 2024. Recruitment followed the same procedures as the first round. The interview guide included screenshots to demonstrate features of SALT and questions on how the current tool could inform decisions, potential improvements in visualization or explanation, and additions to support better decision-making (Multimedia Appendix 1). Sessions were conducted, and data were analyzed using the methods described earlier.

Insights generated from these focus groups were used to summarize the usability and effectiveness of SALT. We also compiled and summarized general suggestions for future development of SALT.

### Ethical Considerations

This work did not involve engagement with human subjects research by the academic investigators as defined under US federal regulations (45 CFR 46). Participant recruitment, focus group facilitation, and qualitative data analysis were conducted by a professional research firm. The academic investigators did not participate in recruitment, did not interact with participants in the context of the focus groups, and did not have access to identifiable or raw qualitative data, receiving only anonymized, aggregated thematic findings. Although the study population comprised professionals with whom the investigators may have had prior or concurrent professional interactions unrelated to this study, no investigator-participant interaction occurred for the purposes of data collection or analysis. Accordingly, institutional review board approval for the academic investigators was not required.

## Results

### Design Focus Groups

We engaged approximately 20 individuals from 9 Greater Cincinnati public health and EMAs (Cincinnati Health Department, Hamilton County Public Health, Butler County Public Health, Clermont County Public Health, Highland County Public Health, Hamilton County Emergency Management, Butler County Emergency Management, Clinton County Emergency Management, and Warren County Emergency Management) in guided question-and-answer discussions to guide the development of SALT. Those participating in these initial design focus groups identified several themes related to data elements, sources, and information that they used or would find useful for situational awareness and learning in future public health events (Table 1). The groups emphasized that multiple individuals and organizations received various data forms, necessitating the need for multiple dashboards tailored to specific audiences and uses. Effective data management required integrating data from diverse sources, such as health information exchanges, state vaccine registries, and local health care facilities, which highlighted the need for preestablished legal and infrastructure frameworks to facilitate timely data sharing. This need became especially evident as many of the emergency data connections established during the pandemic dissolved once the emergency declarations ended, leading to delays in responsive actions due to the time required to secure new data sources and legal agreements.

**Table 1.** Design requirements for the situational awareness learning tool (SALT), distilled from feedback generated during design focus groups of leaders from various agencies and organizations involved in the pandemic response in counties contained in the Ohio Hospital Association’s COVID-19 Zone 6, including city and county public health departments, hospitals, and emergency management agencies.

Requirement	Description
Data compilation	Data should be compiled from multiple sources across the health care spectrum and beyond and submitted to a centralized host where data can be matched, geocoded, aggregated, and analyzed.
Legal framework	The legal framework for sharing data through SALT should reflect use cases and appropriate security and enable the wider use of sensitive, proprietary, or nonopen data.
Non–health care data	Data from regional non–health care organizations, such as schools and wastewater surveillance, are needed for informed decision-making.
Tools and dashboards	Tools and dashboards should be tailored to specific roles and audiences, with at least 2 levels: high level for community- or region-based action and detail view for person-, neighborhood-, or census tract–based intervention.
Update frequency	Updates should be made at least weekly for high-level reporting and more frequently for detailed reporting.
Surveillance measures	Surveillance measures to include and trend over time should cover infection rates, testing counts and rates, hospitalization counts and rates, mortality counts and rates, long-term care facility infection counts and rates, students in quarantine counts and rates, viral shed from wastewater surveillance rates, hospital staffing counts, and vaccination rates.
Disaggregation	The ability to disaggregate regional measures by demographics and social determinants of health would be beneficial for targeting resources and understanding potentially inequitable outcome distributions.
Accessibility	Electronic media and dashboards should be 508 compliant and accessible to users with disabilities.

The discussions also addressed the application of data in decision-making, underscoring the importance of having clear, timely, and geographically relevant data to guide the allocation of scarce resources such as personal protective equipment, laboratory kits, and human resources. Visualizations that included trend forecasting and clear action thresholds proved vital for planning and resource allocation, helping to define when and where to deploy resources based on set criteria. Moreover, the need for a centralized data hub became apparent, with frustrations voiced over the labor-intensive nature of data compilation and the inefficiency of the existing disparate systems. Participants described the ideal data tool as intuitive, tailored to specific user needs, and capable of providing real-time, actionable insights with minimal user manipulation required, ensuring rapid response capabilities in future emergencies.

One theme underscored that the perceived relevance and usefulness of data and information depend on the audience and the purpose of the tool or dashboard that displays them. Another theme was that access to data from multiple sources is essential, but it requires a legal and infrastructure framework that supports data sharing and integration before a crisis occurs. The participants also emphasized that data and information should inform decision-making and resource allocation based on identified needs, trends, forecasts, and thresholds. Furthermore, participants highlighted the importance of data visualizations that are clear, intuitive, and actionable, with definitions and annotations to avoid misinterpretation. Additionally, they stressed the need for data sharing among different groups (eg, schools and hospitals) for coordinated and effective response. Moreover, they expressed the challenge of compiling and interpreting data, which can be resource intensive and time consuming, and suggested the need for a singular, trustworthy data feed that reduces the burden and complexity of data analysis. Finally, they pointed out the gap between data received and action to take, which was often lagging and inefficient, even as events on the ground called for timely and near-real-time data.

Focus group participants identified many sources of data used during the COVID-19 pandemic. All group sessions independently identified that additional data sources may have been used; however, some sources could not immediately be recalled during the focus group sessions as they may have been used for short-term analysis or transitioned to alternate sources. Among the data sources cited were health information exchanges for COVID-19 laboratory testing, admission, and discharge data; publicly available data sources such as the Centers for Disease Control and Prevention, World Health Organization, and the New York Times; water treatment facilities and sewage data with COVID-19 viral testing; and public school reporting with quarantine and isolation status. Data were also drawn from personal protective equipment inventories, including hospitals, health care providers, community organizations, and EMAs; long-term care facilities; skilled nursing facilities; Ohio Department of Health; Ohio Hospital Association; Greater Dayton Area Hospital Association; Ohio Disease Reporting System; Empower Data, which identifies residents dependent or homebound with medical devices; and vaccination registries.

### Platform Development

To optimize the flexibility and real-time capabilities of our system, we chose to use a commercial platform designed for code-based analytical content sharing and management. This platform supports the development of interactive, data-driven solutions using R (R Foundation) or Python (Python Software Foundation), integrating these applications within a version-controlled environment (Git) to facilitate continuous integration and testing.

SALT has been effectively implemented within a VPC, and the methodology for content publication has demonstrated its effectiveness via its use in regular briefings in the state response structure, including the regional Hospital Steering Committee and the regional Multi-Agency Coalition (health commissioners, hospital leaders, and state EMAs). It is also used in regular meetings of state health-related professional societies and in local and regional meetings of informal physician and nursing groups, including the Skilled Nursing Facility and Hospital Discharge Group. Additionally, SALT has replaced the manual, weekly process of posting a readable document to a website with an automated process.

The SALT landing page serves to introduce the tool and serve as a portal for future collaborations between potential partners and the SALT team. Upon signing in, publishers can manage their version-controlled content and adjust access settings, while authorized viewers can access permitted content.

For user management, we developed an internal dashboard to monitor usage metrics around specific items and publisher activities. This dashboard provides weekly updates on user accounts, content views, and resource use. The data collected are used in a charge-back system designed to offset computing and licensing costs effectively.

### Respiratory Illness Situational Awareness

**Figure 1.**
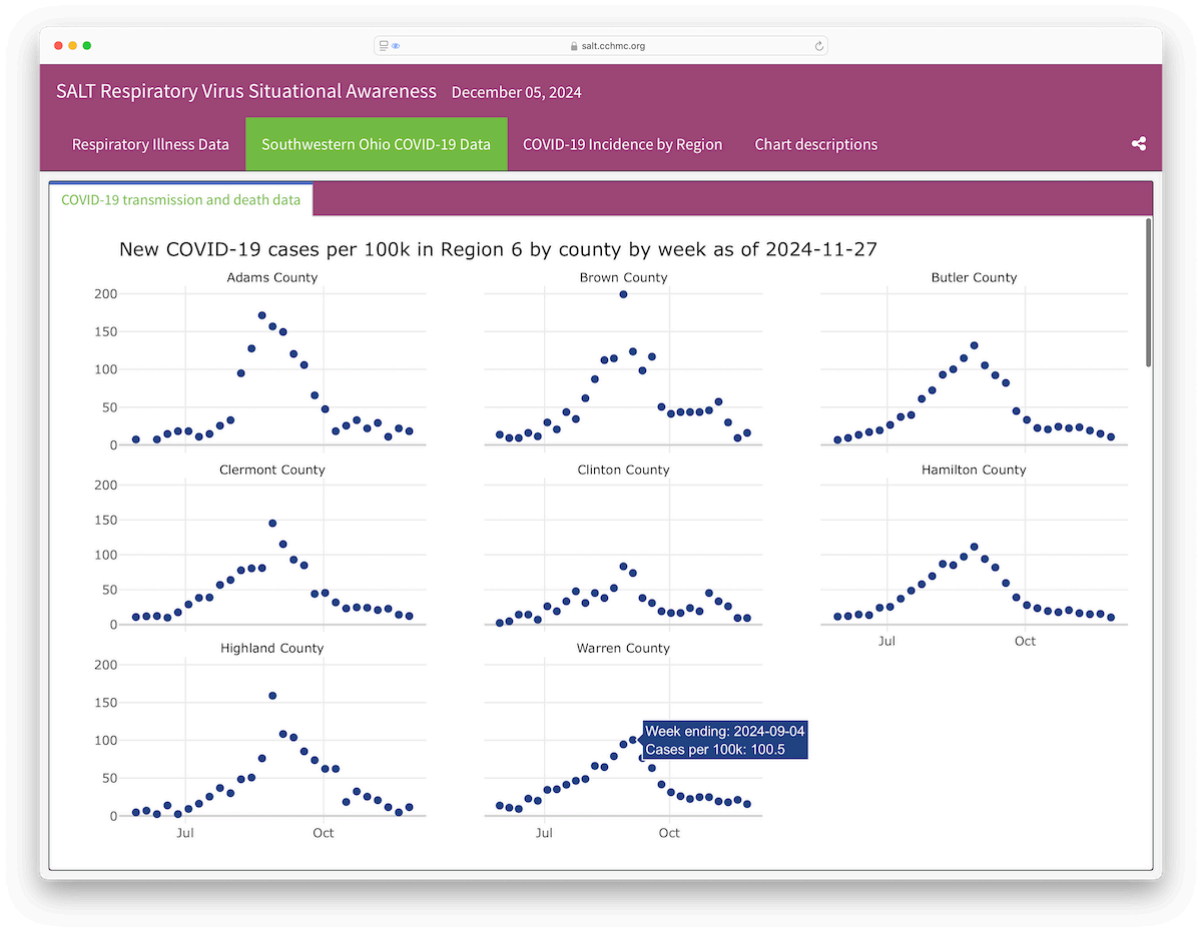
A screen capture of the publicly available SALT respiratory situational awareness dashboard [17]. The currently displayed tab highlights data specific to COVID-19 in southwestern Ohio. SALT: situational awareness and learning tool.

A notable feature available to the public is the Greater Cincinnati COVID-19 dashboard (Figure 1). Previously a collection of static files and images, this publicly available dashboard is now hosted online [17], featuring interactive visuals and a contemporary tabbed layout. It has become an instrumental resource for regional hospital executives and senior public health administrators, aiding in effective resource management and enhancing care delivery across the region.

The regional Hospital Steering Committee, founded early in the COVID-19 pandemic, continues to meet virtually periodically and uses SALT for situational awareness of community respiratory disease. The Hospital Steering Committee dashboard is offered as a distinct and separate area on SALT because it is purpose designed to provide this user group with specific information according to a standardized narrative structure. The committee selected this narrative structure for briefings early in the COVID-19 pandemic in which a sequence of data was presented first at the national level, followed by state-level data, followed by regional- and local-level data. In this way, users gain both a sense of awareness of disease activity locally and of threats in other places that may materialize in the local region of interest. Authorization restricts viewership of data that cannot, due to sensitivity or regulatory reasons, be shared with non–Steering Committee SALT users. This area is frequently used to present brief updates in virtual meetings; committee members can also check the dashboard for updates between committee meetings.

### Evaluation Focus Groups

The aggregated discussion themes and key elements from the focus groups highlight the perceived utility and areas for improvement of the SALT (Table 2). Participants affirmed SALT’s potential effectiveness in managing new respiratory illness pandemics, noting its role as a reliable, consistent, and timely information source that could enhance decision-making and strategic planning, particularly with its ability to track infection rates and hospital occupancy rates, and identify vulnerable populations. Key feedback emphasized the need for more efficient resource allocation, with suggestions to include more specific analytics, such as medical equipment data, to improve actionable outcomes. Moreover, the focus groups suggested enhancements to the tool’s usability through better visualization clarity, narrative explanations, and the incorporation of additional data elements, such as the availability of personal protective equipment and more granular geographic details. Recommendations also covered the integration of predictive analytics and sector-specific data to broaden SALT’s applicability and enhance its utility in public health management.

**Table 2.** Improvements for the situational awareness learning tool, distilled from feedback generated during design evaluation focus groups of leaders from various agencies and organizations involved in the pandemic response in counties contained in the Ohio Hospital Association’s COVID-19 Zone 6, including city and county public health departments, hospitals, and emergency management agencies.

Improvement	Description
Clearer data labeling and improved visual design	Add more descriptive titles, axes labels, and consistent naming conventions for data elements.
Expanded data coverage	Incorporate PPE^a^ and vaccine inventory data and allow for more granular geographic reporting (eg, ZIP code or census tract level).
Narrative explanations	Include short narrative descriptions to accompany key graphs and charts for nontechnical users.
Forecasting capabilities	Add short-term predictive models for health system strain, particularly for ICU^b^ and emergency department usage.
Legal framework for secure data sharing	Ensure the infrastructure is in place for real-time, multisector data integration before a crisis arises.

a PPE: personal protective equipment.

b ICU: intensive care unit.

## Discussion

### Principal Findings

Overall, we achieved our objective to build and maintain a capability that enables data sharing, rapid analysis, and situational awareness in support of the health and well-being of the regional population, both at present and in potential future pandemics or related crises. This is embodied in the cloud-based SALT, which is capable of rapidly and flexibly supporting collaboration, receiving and sharing data, analyses, and communications with and between partners at different levels of security and access.

The relevance of these core ideas was vetted with a diverse set of potential end users, who collectively expressed the need for such a system. These users described a set of system requirements that SALT designers used to select the technical solutions described in this report. For example, as described in Table 1, the user focus groups found that it is useful if data can be compiled from multiple sources; dashboards and analytic products can be tailored to specific audiences in accordance with appropriate levels of security; and updates to products can be made when data change. SALT supports these and other user requirements.

Situational awareness using appropriate, multisource data is a critical component in managing public health emergencies such as pandemics [13]. It involves the perception of environmental elements, comprehension of their meaning, and projection of their status in the near future [18]. Experience in the COVID-19 pandemic illustrated that situational awareness is essential for effective, timely public health decision-making and responses [2,8,9,19].

Moreover, SALT uses open-source scripting languages (R and Python), so that scripts generating specific analytic products can easily be shared. Over time, SALT will accumulate a library of scripts that will reduce future product development time. Similarly, user-requested revisions to analytic products can easily be accommodated at the script level, which is not always possible using commercial, proprietary visualization software.

Centralized deployment of analytic content using script-based sources and processes also facilitates the monitoring of the SALT to collect and analyze usage data to continuously improve user experiences.

The ability to segment access to data according to SALT user credentials allows data to be shared easily and rapidly with those who have a validated need and permission. Nevertheless, data use agreements are still needed to access and share protected data. Given recent instabilities in the federal public health and demographic data ecosystems [20,21], it is critical for jurisdictions to begin establishing data sharing agreements across multiple health and social sector actors so that tools such as SALT can be used for situational awareness.

While SALT is still in the early stages of use and engagement, a successful launch, feedback gathering, and content deployment have been achieved. These benchmarks comprise the foundation that SALT will continue to build upon. The technical architecture and systems developed here have proven to be capable of accomplishing the objective of SALT itself, that being an effective and efficient situational awareness and learning system presenting near-real-time analytic content available to public health professionals.

A potential limitation of our broader contribution is that SALT was developed for Southwest Ohio, although the challenges it addresses are not unique to our region. The COVID-19 pandemic revealed limitations in how public health data are collected, integrated, and shared across the nation [9]. For example, local communities faced, and continue to face, similar barriers in accessing timely, multisource data; managing legal and privacy frameworks; and tailoring dashboards to different audiences. Our work demonstrates how we overcame such barriers. While the SALT pilot work occurred in Ohio, these insights are widely applicable and provide a roadmap for other jurisdictions and organizations to strengthen situational awareness and preparedness for pandemics and complex health challenges.

### Conclusions

While an upcoming period of expansion and scaling will continue to evaluate the effectiveness of the tool, the systems and architecture are established and ready for widespread adoption, implementation, and success. Beyond the application described in this report related to pandemics, SALT is applicable to other health-related and health care–related domains. Learning health networks, which collect and transform multisource data into situational awareness for a range of users, including clinicians, patients, and caregivers, are but one example. A range of community health issues require comprehensive situational awareness for decision-making, such as programs improving child and maternal health equity and projects aimed at reducing asthma-related hospitalizations. It is our hope that the work described herein can and will be applied to the spectrum of health-related applications requiring shared situational awareness.

## Supplementary material

10.2196/77379 Multimedia Appendix 1 Questions used in design and evaluation focus groups.

## Abbreviations

CFR: Code of Federal Regulations
EMA: emergency management agency
SALT: situational awareness and learning tool
VPC: virtual private cloud
