# Supplementary material for: Building Enhanced Public Health Data Systems With a Situational Awareness and Learning Tool: Focus Group Study
Source: Online J Public Health Inform. 2026 Apr 29;18:e77379. doi: 10.2196/77379 (PMC13128156; doi:10.2196/77379)
Supplement: Multimedia Appendix 1 [file ojphi-v18-e77379-s001.docx]

**Appendix**

Questions used in Design Focus Groups:

1. Did anyone in your organization receive data during the pandemic?
   1. What roles received data?
   2. Did everyone who needed it have direct access?
   3. Do you remember the data source (Ohio Department of Health, Emergency Mangement Agency, Cincinnati Children’s, The Health Collaborative, Ohio Hospital Association, etc.)?
2. What decisions were informed by data during the pandemic?
   1. How often were data reviewed?
   2. What data or variables were helpful?
   3. What data did you wish you had, but didn’t?
3. What type of geographic data was helpful?
4. Did trend data inform decisions?
5. What frustrated you about available data?
6. Describe the ideal data tool or dashboard in a future emergency
   1. What questions would it answer?
   2. How would it help you do your job?
   3. What data elements are included?
   4. Who has access?
   5. How often are you accessing it?

Questions used in Evaluation Focus Groups:

1. What decisions could be informed by the data currently in the tool?
   1. What questions would this tool have answered if it were available at the start of the previous COVID-19 pandemic?
   2. Would this tool, as demonstrated, improve the speed or confidence in your decision making compared to the start of the COVID-19 pandemic and today? How?
2. What could be visualized or explained differently?
   1. Were the charts, graphs, and tables intuitive or immediately understandable? Or loaded with unnecessary details or not loaded with enough details?
3. What can be added to assist in decision making?
   1. What is missing?
   2. What additional questions would it answer?
   3. How would adding new elements help you do your job?
   4. How does the tool compare to other tools you’ve used for situational awareness?
   5. What other issues could this tool be used/developed to assist in decision making?
